# *Toxoplasma gondii* infection and toxoplasmosis in North Africa: a review

**DOI:** 10.1051/parasite/2019006

**Published:** 2019-02-15

**Authors:** Mariem Rouatbi, Safa Amairia, Yosra Amdouni, Mohamed Anis Boussaadoun, Ouarda Ayadi, Amira Adel Taha Al-Hosary, Mourad Rekik, Rym Ben Abdallah, Karim Aoun, Mohamed Aziz Darghouth, Barbara Wieland, Mohamed Gharbi

**Affiliations:** 1 Laboratoire de Parasitologie, Univ. Manouba, École Nationale de Médecine Vétérinaire de Sidi Thabet 2020 Sidi Thabet Tunisia; 2 Université des Frères Mentouri, Constantine 1, Institut des Sciences Vétérinaires Route de Batna El Khroub 25100 Constantine Algérie; 3 Department of Animal Medicine (Infectious Diseases), Faculty of Veterinary Medicine, Assiut University 71526 Assiut Egypt; 4 International Center for Agricultural Research in the Dry Areas (ICARDA) P.O. Box, 950764 Amman 11195 Jordan; 5 Laboratoire de Parasitologie Médicale, Biotechnologie et Biomolécules, Institut Pasteur de Tunis, Université Tunis El Manar BPO 74 1002 Tunis Tunisia; 6 International Livestock Research Institute (ILRI) P.O. Box 5689 Addis Ababa Ethiopia

**Keywords:** *Toxoplasma gondii*, North Africa, Humans, Animals

## Abstract

Toxoplasmosis is an important zoonosis caused by an obligate intracellular parasitic protozoan, *Toxoplasma gondii*. The disease is distributed worldwide and can affect all warm-blooded vertebrates, including humans. The present review aimed to collect, compile and summarize the data on the prevalence of *T. gondii* infection in humans and animals in the five North African countries (Morocco, Algeria, Tunisia, Libya and Egypt). Published data from national and international databases were used. Distribution patterns and risk factors for *T. gondii* infection are discussed, focusing on biotic and abiotic factors. This review is a comprehensive epidemiological analysis of *T. gondii* infection in North Africa and will therefore be a useful tool for researchers. It can also be used to propose or enhance appropriate national toxoplasmosis control programs.

## Abstract (fr)

La toxoplasmose est une zoonose importante causée par un protozoaire parasite intracellulaire obligatoire, *Toxoplasma gondii*. La maladie est répandue dans le monde entier, chez tous les vertébrés à sang chaud, y compris les humains. La présente étude visait à collecter, compiler et résumer les données sur la prévalence de l’infection par *T. gondii* chez l’homme et les animaux dans les cinq pays d’Afrique du Nord (Maroc, Algérie, Tunisie, Libye et Égypte). Les données publiées dans des bases de données nationales et internationales ont été utilisées. Les schémas de distribution et les facteurs de risque d’infection par *T. gondii* sont discutés, en se concentrant sur les facteurs biotiques et abiotiques. Cette synthèse est une analyse épidémiologique complète de l’infection par *T. gondii* en Afrique du Nord et sera donc un outil intéressant pour les chercheurs. Elle peut également être utilisée pour proposer ou renforcer des programmes nationaux appropriés de contrôle de la toxoplasmose.

## Abstract (ar)

داء المقوسات هو مرض مشترك هام يسببه طفيلي وحيد الخلية اجبارى داخل خلوى، توكسوبلازما غوندي ( *Toxoplasma gondii *). تنتشر هذه الاصابة في جميع أنحاء العالم وعند جميع الفقريات ذوات الدم الحار بما في ذالك الانسان. تهدف هذه الدراسة الببليوغرافية إلى جمع وتصنيف وتلخيص البيانات الحالية حول نسبة الاصابة بالطفيلي توكسوبلازما غوندي ( *Toxoplasma gondii *) لدى الانسان والحيوان في بلدان شمال إفريقيا الخمسة (المغرب والجزائر وتونس وليبيا ومصر) .تم استعمال المعلومات المنشورة بقاعدات البيانات الوطنية و العالمية كما تمت مناقشة أنماط التوزيع وعوامل خطر الاصابة بالطفيلي توكسوبلازما غوندي ( *Toxoplasma gondii *) مع التركيز على العوامل الحياتية و الغير حياتية.

تمثل هذه الدراسة تحليلاً وبائياً شاملاً للإصابة بالطفيلي توكسوبلازما غوندي ( *Toxoplasma gondii *) في شمال إفريقيا. وبذلك فإن هذه الدراسة هي أداة مفيدة للباحثين. كما يمكن استعمالها كي يتم اقتراح او تعزيز برامج وطنية مناسبة لمكافحة داء المقوسات.

## Introduction

*Toxoplasma gondii* was discovered in North Africa, more precisely in the Pasteur Institute of Tunis. In fact, during experiments on leishmaniosis, Nicolle and Manceaux observed an arc-shaped protozoan in tissues of a North African rodent, the gundis (*Ctenodactylus gundi*) [40]. It was named *Toxoplasma gondii* (*T. gondii*) based on its morphology (*Toxon*: arc, *plasma*: form) and its host.

The protozoan *T. gondii* is the agent of toxoplasmosis. It infects all warm-blooded animals including birds and mammals [41]. Toxoplasmosis is an important health problem worldwide [119]. The history, the epidemiological status, the life cycle, and the development of this parasite have been well studied around the world [40, 98, 126].

North Africa, a south Mediterranean region, lies between Sub-Saharan Africa and Europe. It represents a specific agro-ecological and socio-economic context, leading to specific epidemiological patterns for several human and animal diseases. The past and current status of *Toxoplasma* infection in North African countries is not well understood since few studies are available for the whole region.

This review aimed to collect, update and analyse the epidemiological data on *Toxoplasma* infection in five North African countries (Morocco, Algeria, Tunisia, Libya and Egypt), where several studies have been published in the grey literature but are not available to international readers.

## General geographical context

North Africa includes five countries: Morocco, Algeria, Tunisia, Libya and Egypt (Table 1). All countries have a predominantly semi-arid to arid bioclimate with large desert areas covering more than 75% of the region, mainly in the centre and southern parts. The summer season is hot and dry. The rainy season is from October to April with maximum precipitation from December to February [55, 88]. High variability of inter-annual precipitation is observed. Rain is scarce in the Sahara, where temperatures reach up to 55 °C during the day and drop to below 0 °C at night.

**Table 1:** Characteristics of North African countries.

Country	Capital city	Area (km^2^)	Sahara surface (km^2^)	Population (2017)	Density (inhabitants/km^2^ in 2017)	Percentage of total world population (%)	Percentage urban population (2017) (%)	Median age (years)
Morocco	Rabat	446,300	–	35,913,182	80	0.47	59.6	28.3
Algeria	Algiers	2,381,740	2,000,000	41,582,461	17	0.55	73	27.8
Tunisia	Tunis	155,360	90,000	11,580,938	74	0.15	66.9	31.4
Libya	Tripoli	1,759,540	700,000	6,411,555	4	0.08	80.3	27.6
Egypt	Cairo	995,450	3,000,000	98,250,741	98	1.29	38.8	24.8

Source: Worldmeters [127].

Based on the latest United Nations estimates, the current population of North Africa is 192,517,616 as of November 2017 [127] (Table 1). The most heavily populated area is the coastal strip because of its fertility and mild weather.

In North African cuisine, the most common staple foods are fish, seafood, goat meat, lamb, beef, dates, almonds, olives, various vegetables, and fruits. Because the region is predominantly Muslim, pork is not consumed, and animals are totally bled when slaughtered. Meat is predominantly consumed cooked in sauce, but undercooked grilled lamb is consumed during the Muslims’ sacrifice feast, and very often in restaurants at the side of the road [125].

Livestock (cattle, buffaloes, camels, sheep, goats, and poultry) play an important role in food security, nutrition, and the economies of North African countries by supporting rural livelihoods and employment, and ensuring access to animal source foods (ASF) [58]. In the near East and North Africa, consumption of ASF has risen by 4% over the past two decades to reach 13.4 million tonnes for meat in 2014 and 35 million tonnes (in milk equivalents) for milk and dairy products (Table 2) [58].

**Table 2:** Livestock indicators in North Africa, 2014.

Country	Livestock/TLU (2013)	Livestock as share of gross value of agricultural production (constant 2004-2006 US$)	Total meat production (1000 tonnes)
Morocco	4,897,310	40%	1,077
Algeria	4,829002	35%	681
Tunisia	1,499,680	24%	320
Libya	1,176,450	NA	176
Egypt	7,527,000	43%	1,810

Source: These data were compiled from different sources [58].

TLU: Tropical Livestock Units.

NA: Not Available.

## Life cycle of *Toxoplasma gondii* in the North African context

*Toxoplasma gondii* is an obligate apicomplexan intracellular protozoan; it has a cosmopolitan distribution [106]. The life cycle of *T. gondii* involves (i) felines, essentially domestic cats, as definitive hosts in which sexual reproduction occurs, and (ii) intermediate hosts, where asexual reproduction occurs; the latter consist of all warm-blooded animals, including birds and mammals, with *T. gondii* being most common in sheep [120]. Felines can host both sexual and asexual reproduction and are also referred to as integral hosts. The infective stages of *T. gondii* consist of three forms: (i) tachyzoites present during the early infection period, (ii) bradyzoites present in the intermediate hosts as tissue cysts, and (iii) sporulated oocysts containing sporozoites, shed as non-sporulated oocysts by the final hosts with feces [43]. In North Africa, there are seven species of wild felids that may be involved in the life cycle of *T. gondii* (Table 3). To the best of our knowledge, the population of domestic cats in North Africa has never been estimated even though it is reported to be very high, especially in urban areas.

**Table 3:** Wild felid species present in North Africa.

Species	Description	Geographic distribution	Reference
Caracal	Body length: 61–105 cm	Mauritania, Morocco,	[89]
*Caracal caracal* Schreber, 1776	Height: 40–50 cm	Algeria, Tunisia, Libya,	
	Weight: 8–20 kg	Egypt	
	In captivity, average lifespan as long as 16 years		
		
Chaus	Relatively short tail, long legs, big	Morocco, Algeria, Egypt	[95]
*Felis chaus* Schreber, 1777	pointed ears		
		
Sand cat	Weight: 2–3 kg	Morocco, Algeria,	[100]
*Felis margarita* Loche, 1858	Living in arid areas with temperatures ranging from 0 °C to 58 °C	Tunisia, Libya, Egypt	
	
Serval	Medium sized African cats	Morocco, Algeria, Tunisia	[89]
*Felis serval* Schrever, 1776	Body length: approximately 60 cm		
	Weight: on average 14 kg		
	Average life in the wild is 10 years		
African wildcat	Body length: 40–66 cm	Morocco, Algeria,	
*Felis silvestris lybica* Forster, 1780	Tail length: 24–37 cm	Tunisia, Libya, Egypt	
	Weight: 2.4–6.4 kg		

Transmission of the infection to humans occurs through three main routes: (i) ingestion of oocysts of *T. gondii* shed by felids [41, 102], (ii) ingestion of tissue bradyzoites in undercooked or raw infected meat, and (iii) vertical transmission across the placenta from the mother to the fetus [90, 120]. If the parasite is contracted for the first time during pregnancy, it may be transmitted to the fetus [120]. This vertical or congenital transmission could result in the invasion of the placenta by tachyzoites which may cross the placenta and enter fetal tissues or the bloodstream [99]. Congenital toxoplasmosis may cause abortion, neonatal death, or fetal abnormalities mainly in the neuromuscular system and eyes [70, 103, 104]. Even though infection with *T. gondii* is very common in humans, clinical signs are uncommon in immunocompetent people. In risk groups such as immunocompromized persons and newborns with congenital infection, clinical signs such as encephalitis, pneumonia and ophthalmologic disorders can occur [120]. *Toxoplasma gondii* infection can also rarely be transmitted by tissue or organ transplants [106].

In pregnant animals, primary infection can lead to abortion, hence causing high economic losses [22]. In ewes, if the infection occurs between 50 and 120 days of pregnancy, it induces abortion, expulsion of mummified fetuses, or the birth of stillborn and weak lambs. After 120 days of pregnancy, the infection generally leads to apparently normal lambs that can survive for a few days or grow normally and become protected against re-infections [23].

Toxoplasmosis in rabbits and poultry has not been well studied; nevertheless, these two species represent a potential source of *T. gondii* infection [122]. Transplacental transmission of *T. gondii* has been reported in rabbits since more than 40 years [122]. Clinical toxoplasmosis in rabbits is apparently rare and not specific [38, 117].

Biotic and abiotic factors play important roles in *T. gondii* transmission and thus in the epidemiology of *T. gondii* infection. These factors determine host geographic distribution, density, and interactions [120]. Temperate areas with sufficient rainfall located in the coastal area and the Atlas mountains of North Africa are the most favorable for the survival and spreading of oocysts shed by the definitive hosts. In fact, if the temperature and hygrometry are high, the viability of the oocysts increases, leading to higher contamination rates of intermediate hosts [128]. In such areas, the number of different herbivore species is also high, creating further favorable conditions for *T. gondii* transmission (Table 4).

**Table 4:** Estimated domestic herbivore population in North Africa (1000 heads).

Country	Sheep	Goats	Cattle	Camels	Equines
Morocco	17,078	5118	2814	70	NA
Algeria	20,000	3800	1650	290	218
Tunisia	7616	1550	1400	200	187
Libya	4500	1265	130	47	NA
Egypt	2258	1054	2810	68	1072
Overall	60,302	18,387	10,494	2275	NA

NA: Not Available.

Source: OIE [97] and FAO [57].

## Main findings of the surveys carried out in North Africa

A literature review on the seroprevalence and the molecular prevalence of *T. gondii* among human and animals in North African countries was conducted. Publications related to *T. gondii* infection and toxoplasmosis in North Africa were collected from two literature databases including PubMed and Google Scholar. Keywords used for the bibliographic search were “Morocco, Algeria, Tunisia, Libya, Egypt, human, animal, toxoplasmosis”. No time limitation was imposed and the search took place in 2015, with an update in 2018. The selected articles respected six criteria: (i) study was performed in humans and animals from five North African countries; (ii) both serologic and molecular techniques were considered; (iii) only natural infection by *T. gondii* was taken into consideration; (iv) studies carried out with vaccine assays were not taken into consideration; (v) in each country, information regarding prevalences of infection by *T. gondii* were organized by species, starting with humans then animals; and (vi) only articles written in English and French were considered.

### *Toxoplasma gondii* infection in Morocco

All investigations carried out in Morocco in both humans and animals were based on serological tests using enzyme-linked immunosorbent assay (ELISA). The seroprevalence of *T. gondii* infection in humans was studied for the first time in 1969 by Le Viguelloux and Epardeau [87]. A high infection rate of 64.9% was reported using an indirect immunofluorescence test. Shortly after, the same test was applied for the detection of *T. gondii* antibodies in 1,026 human sera from Rabat city [96]. Since then, congenital toxoplasmosis has been the main issue in published papers in Morocco. Using the same serological test (ELISA), the seroprevalence in pregnant women ranged between 36.7% and 62.1%, between 2007 and 2017 (Table 5). As a novel diagnostic tool, the chemiluminescent microparticle immunoassay (CMIA) was used for *T. gondii* antibodies detection among pregnant women in Fes city [121]. Among the risk factors, age was the most commonly reported factor in these studies and the overall conclusion is that the prevalence of *Toxoplasma* infection increases with age [17, 83–85]. Infection rates also varied according to the locality; reaching 50.6% in Rabat which is higher than 43.3% in Nador (North East), 42.6% in Tetouan (North) and 36.7% in Kenitra (North West) [52]. The authors attributed this difference to the temperate climate of Rabat city, which maintains the biological cycle of *T. gondii* (rapid and complete sporulation). Regular contact with the land (soil, gardening and agricultural activities) was retained as a major risk for *T. gondii* infection in Rabat city [52, 85]. In one study conducted in Rabat and concerning pregnant women, school level and knowledge of toxoplasmosis modes transmission were found to be risk factors (*p* < 0.01), while the consumption of raw meat, contact with cats, and level of hygiene were not significant. Toxoplasmosis was also studied in HIV-infected patients in the city of Marrakech and its surroundings [1]. The authors studied the seroprevalence of *T. gondii* in 95 HIV-infected adults of different ages. Seroprevalence was estimated to be 62.1%.

**Table 5:** Human toxoplasmosis prevalence in North African countries.

Country (author)	Region	Population/sample	Technique	Positive/examined (%)	Reference
Morocco (Le Viguelloux and Epardeau, 1969)	–	Patients	IFAT^a^	100/154 (64.9)	[87]
Morocco (Nejmi and Alami, 1973)	Rabat	Military personnel, schoolgirls and pregnant women	IFAT	281/1026 (27.4)	[96]
Morocco (Biava et al., 1983)	Marrakech	Women	IFAT/Hemagglutination	106/318 (33.3)	[32]
Morocco (Guessous-Idrissi et al., 1984)	–	Women	–	−(51.5)	[65]
Morocco (El Mansouri et al., 2007)	Kenitra	Pregnant women	ELISA^b^	−(36.7)	[52]
Morocco (El Mansouri et al., 2007)	Nador	Pregnant women	ELISA	−(43.3)	[52]
Morocco (El Mansouri et al., 2007)	Tetouan	Pregnant women	ELISA	−(42.6)	[52]
Morocco (El Mansouri et al., 2007)	Rabat	Pregnant women	ELISA	1242/2456 (50.6)	[52]
Morocco (Laboudi et al., 2009)	Rabat	Pregnant women	ELISA	516/1020(50.6)	[85]
Morocco (Barkat et al., 2010)	Rabat	Pregnant women	–	163/368 (44.3)	[17]
Morocco (Addebbous et al., 2012)	Marrakesh	HIV-infected adults	Indirect ELISA	59/95 (62.1)	[1]
Morocco (Laboudi et al., 2014)	Rabat	Pregnant women	ELISA	549/1169 (47)	[84]
Morocco (Laboudi, 2017)	Rabat	Pregnant women	ELISA	59/128 (46.1)	[83]
Morocco (Tlamcani et al., 2017)	Fes	Pregnant women	CMIA^c^	1367/3440 (39.7)	[121]
Algeria (Balozet, 1955)	Algiers	Humans	CFT^d^	13/125 (10.4)	[16]
Algeria (Schneider et al., 1977)	Algiers	Patients	IFAT	1297/2438 (53.2)	[110]
Algeria (Messserer et al., 2014)	Annaba	Pregnant women	Microparticle enzyme	491/1028 (47.8)	[93]
Algeria (Berredjem et al., 2017)	Annaba	Pregnant women	ELISA	57/143 (39.9)	[31]
Algeria (Berredjem et al., 2017)	Annaba	Pregnant women	PCR^e^ (B1 gene)	9/57 (15.8)	[31]
Algeria (Berredjem et al., 2017)	Annaba	Pregnant women	PCR (P30 gene)	4/14 (28.6)	[31]
Tunisia (Ben Rachid and Blaha, 1970)	–	Blind children	SFDT^f^	19/92 (20.6)	[30]
Tunisia (Ben Rachid and Blaha, 1970)	–	Adolescents	SFDT	18/30 (60)	[30]
Tunisia (Messedi-Triki et al., 1982)	Tunis	–	–	402/810 (49.6)	[92]
Tunisia (Jemni et al., 1985)	Sousse	Students	–	−(67)	[75]
Tunisia (Bchir et al., 1992)	Monastir	Pregnant women	ELISA	195/478 (40.8)	[19]
Tunisia (Ben Ayed Nouira et al., 1994)	Tunis	Women	–	−(63.5)	[27]
Tunisia (Bouratbine et al., 2001)	Beja	Individuals	ELISA and IFAT	830/1421 (58.4)	[35]
Tunisia (Sellami et al., 2010)	Sfax	Pregnant women	ELISA	15952/40567 (39.3)	[111]
Tunisia (Ben Abdallah et al., 2013)	Tunis	Pregnant women	ELISA	944/2070 (45.6)	[26]
Tunisia (Fakhfakh et al., 2013)	Tunis	Pregnant women	Immunocapture	1114/2351 (47.4)	[56]
Tunisia (Siala et al., 2014)	Tunis	Amniotic fluid	PCR	12/60 (20)	[115]
Libya (Khadre and Nageh, 1987)	Tripoli	Adult males	–	1032/2000 (51.6)	[78]
Libya (Khadre and Nageh, 1987)	Tripoli	Adult females	–	130/300 (43.3)	[78]
Libya (Khadre and Nageh, 1987)	Tripoli	Schoolchildren	–	865/1980 (43.7)	[78]
Libya (Khadre and Nageh, 1987)	Tripoli	Female patients with abortion history	–	1334/1921 (69.4)	[78]
Libya (Kassem and Morsy, 1991)	Benghazi	Pregnant women	IHAT^g^	176/369 (47.7)	[77]
Libya (Elsaid et al., 2014)	Tripoli	Control volunteers	ELISA	3/300 (1)	[53]
Libya (Elsaid et al., 2014)	Tripoli	Psychiatric patients	ELISA	151/300 (50.3)	[53]
Libya (Elsaid et al., 2014)	Tripoli	Control volunteers	Latex	140/300 (46.7)	[53]
Libya (Elsaid et al., 2014)	Tripoli	Psychiatric patients	Latex	185/300 (61.7)	[53]
Libya (Gamal and Jaroud, 2015)	Alkhoms	Pregnant women	ELISA	142/361 (39.3)	[60]
Libya (Shalaka et al., 2015)	Tripoli	Patients with HIV/AIDS	–	19/227 (8.4)	[113]
Libya (Gashout et al., 2016)	Tripoli	Women who have had spontaneous abortions	ELISA	54/140 (38.6)	[61]
Libya (Gashout et al., 2016)	Tripoli	HIV patients	ELISA	23/26 (88.5)	[61]
Libya (Gashout et al., 2016)	Sabrata	Patients with leukemia or lymphoma	ELISA	6/9 (66.7)	[61]
Libya (Gashout et al., 2016)	Zawia	Children with ocular infection	ELISA	1/2 (50)	[61]
Libya (Haq et al., 2016)	Misurata	Pregnant women	PCR	27/276 (9.8)	[67]
Egypt (Azab et al., 1992)	–	Serum of lactating women	IFAT	22/70 (31.4)	[13]
Egypt (Azab et al., 1992)	–	Milk of lactating women	IFAT	12/70 (17.1)	[13]
Egypt (Youssef, 1993)	Dakahlia	Inhabitants	Dot-ELISA	−(23.8)	[130]
Egypt (Ibrahim et al., 1997)	Gharbia	Workers	IHAT	11/21 (52.4)	[72]
Egypt (Ibrahim et al., 1997)	Dakahlia	Pregnant women	ELISA	52/101 (51.5)	[72]
Egypt (Amrei et al., 1999)	Zagazig	Children with intellectual disability	–	14/32 (43.7)	[11]
Egypt (Amrei et al., 1999)	Zagazig	Adult females	–	−(37.5)	[11]
Egypt (Elsheikha et al., 2009)	Mansoura	Blood donors	ELISA	155/260 (59.6)	[54]
Egypt (El-Gozamy et al., 2009)	Qualyobia	Pregnant women	ELISA	46.5 to 57.6	[50]
Egypt (Ghoneim et al., 2010)	El Fayoum	Pregnant women	ELISA IgG	27/59 (45.8)	[64]
Egypt (Ghoneim et al., 2010)	El Fayoum	Pregnant women	ELISA IgM	18/59 (30.5)	[64]
Egypt (Ghoneim et al., 2010)	El Fayoum	Pregnant women	SFDT	14/ 59 (23.7)	[64]
Egypt (Ghoneim et al., 2010)	El Fayoum	Pregnant women	PCR	19/59 (33.2)	[64]
Egypt (El Deeb et al., 2012)	Menoufia	Pregnant women	ELFA^h^	218/323 (67.5)	[47]
Egypt (Ahmed et al., 2014)	Sharkia	Pregnant women	MAT^i^	82/100 (82)	[4]
Egypt (Kamal et al., 2015)	Minia	Women with high risk pregnancy	ELISA	61/120 (50.8)	[76]
Egypt (Ibrahim et al., 2017)	Menoufia	Pregnant women	ELISA	63/171 (36.8)	[74]
Egypt (Ibrahim et al., 2017)	Menoufia	Pregnant women	RT-PCR^j^	24/171 (14)	[74]
Egypt (Ibrahim et al., 2017)	Gharbia	Pregnant women	ELISA	60/193 (31.1)	[74]
Egypt (Ibrahim et al., 2017)	Gharbia	Pregnant women	RT-PCR	19/193 (9.8)	[74]

a **IFAT**: ImmunoFluorescent Antibody Test.

b **ELISA**: Enzyme Linked Immunosorbent Assay.

c **CMIA**: Chemiluminescent Microparticle Immunoassay

d **CFT**: Complement-Fixation Test.

e **PCR:** Polymerase Chain Reaction.

f **SFDT:** Sabin-Feldman Dye Test.

g **IHAT:** Indirect Hemagglutination Antibody test.

h **ELFA:** Enzyme-Linked Fluorescence Assay.

i **MAT:** Modified Agglutination Test.

j **RT-PCR**: Real-Time Polymerase Chain Reaction.

**– :** Not Available.

**Figure 1:**
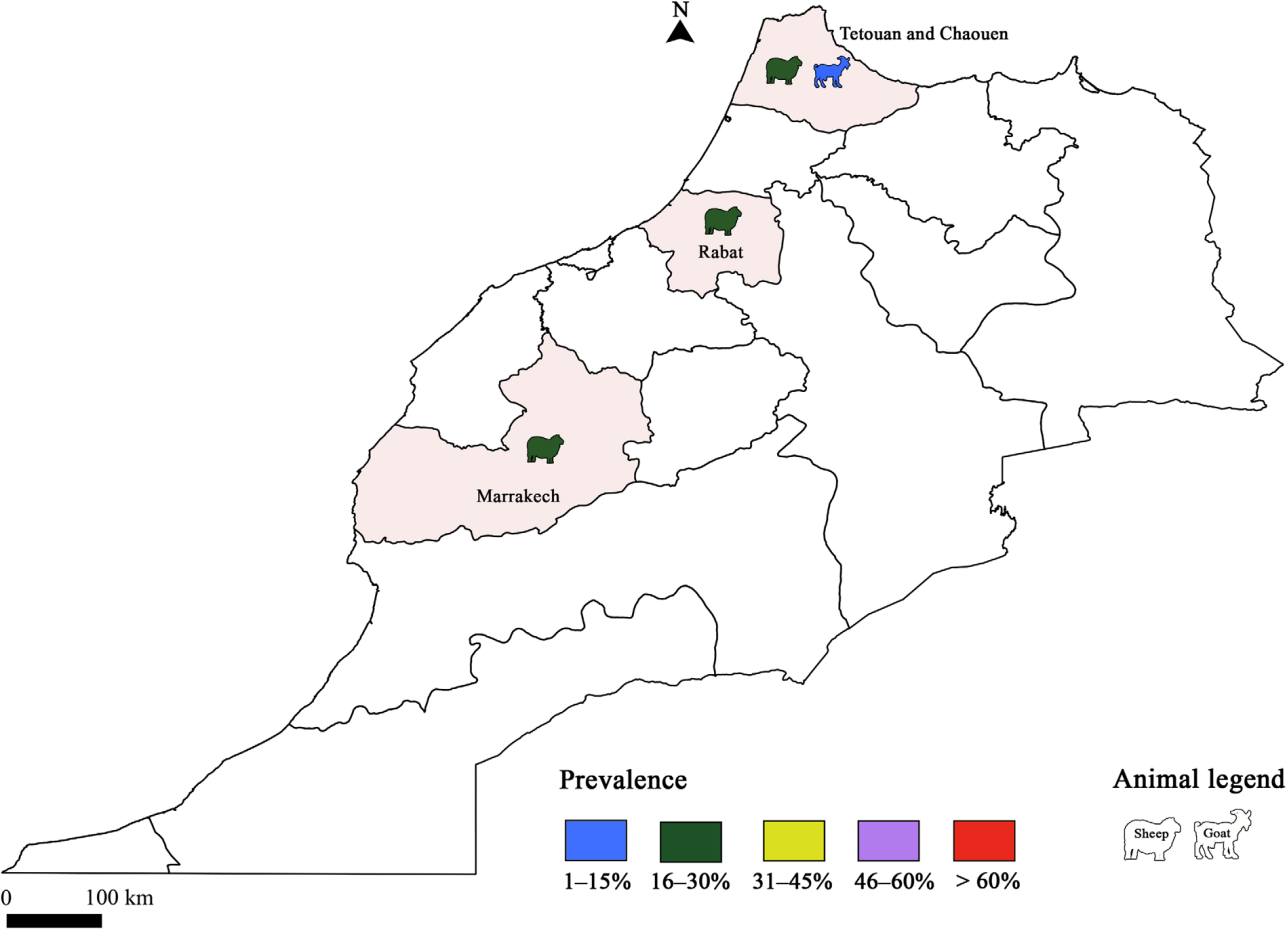
Prevalence of *Toxoplasma gondii* infection in Morocco: Animal toxoplasmosis, seroprevalence.

Few studies have targeted *T. gondii* infection in livestock species; only sheep and goats were concerned (Fig. 1). The seroprevalence of *T. gondii* infection in sheep was lower than 30% using ELISA (Table 6). Using the same diagnostic tool, there was no significant difference according to the locality (30% in Rabat, 27.6% and 30% in the Marrakech region, and 20.8% in Northern Morocco and Middle Atlas) [24, 28, 29, 109]. These similarities could be attributed to herd management and the presence of cats in farms [28]. Sawadogo et al. [109] reported that the infection rate in meat from sheep in the Marrakech region was lower than in other regions. In fact, high temperatures during the summer in Marrakech with an average annual rainfall up to 360 mm could reduce oocyst life span and consequently the prevalence of infection. Determination of *T. gondii* seroprevalence in goats was conducted only in the Northern Morocco and Middle Atlas regions, revealing a low infection rate (8.5%) [29]. One study was performed to determine the genotypes of *T. gondii* occurring in Morocco using 15 microsatellite markers, and referred to a human strain of type III genotype [59].

**Table 6:** Animal toxoplasmosis prevalence in North African countries.

Country (author)	Region	Population/sample	Technique	Positive/examined (%)	Reference
Morocco (Benkirane et al., 1990)	Rabat	Sheep	ELISA	−(30)	[28]
Morocco (Belbacha et al., 2004)	Marrakech	Sheep	ELISA	15/50 (30)	[24]
Morocco (Sawadogo et al., 2005)	Marrakech	Sheep	ELISA	72/261 (27.6)	[109]
Morocco (Benkirane et al., 2015)	Northern Morocco and Middle Atlas	Sheep	ELISA	42/202 (20.8)	[29]
Morocco (Benkirane et al., 2015)	Northern Morocco and Middle Atlas	Goats	ELISA	9/106 (8.5)	[29]
Algeria (Balozet, 1955)	Algiers	Dogs	CFT	32/105 (30.5)	[16]
Algeria (Dechicha et al., 2015)	Blida	Cattle	IFAT	13/332 (3.9)	[37]
Algeria (Dechicha et al., 2015)	Djelfa	Sheep	IFAT	32/276 (11.6)	[37]
Algeria (Dechicha et al., 2015)	Djelfa	Goats	IFAT	14/106 (13.2)	[37]
Algeria (Abdelhadi et al., 2015)	Tiaret	Cattle	ELISA	14/92 (15.2)	[2]
Algeria (Mohamed–Cherif et al., 2015)	Tiaret	Horses	MAT	76/293 (25.9)	[94]
Algeria (Mohamed–Cherif et al., 2015)	Tiaret	Donkeys	MAT	9/30 (30)	[94]
Algeria (Yekkour et al., 2017)	Algiers	Stray cats	MAT	48/96 (50)	[129]
Algeria (Dahmani et al., 2018)	Western, Eastern and South	Sheep	ELISA	48/580 (8.3)	[36]
Tunisia (Ben Rachid and Blaha, 1970)	–	Sheep	SFDT	169/225 (75.1)	[30]
Tunisia (Ben Rachid and Blaha, 1970)	–	Goats	SFDT	51/85 (60)	[30]
Tunisia (Ben Rachid and Blaha, 1970)	–	Cattle	SFDT	93/250 (37.2)	[30]
Tunisia (Ben Rachid and Blaha, 1970)	–	Cattle	SFDT	16/100 (16)	[30]
Tunisia (Ben Rachid and Blaha, 1970)	–	Camels	SFDT	48/120 (40)	[30]
Tunisia (Ben Rachid and Blaha, 1970)	–	Dogs	SFDT	142/200 (71)	[30]
Tunisia (Boughattas et al., 2011)	Sidi Thabet, Monastir and Battan	Horses	MAT	28/158 (17.7)	[33]
Tunisia (Gharbi et al., 2013)	Ben Arous	Sheep	PCR	9/71 (12.7)	[62]
Tunisia (Gharbi et al., 2013)	Kasserine	Sheep	ELISA	35/184 (19)	[62]
Tunisia (Gharbi et al., 2013)	Sidi Bouzid	Sheep	PCR	27/106 (25.5)	[62]
Tunisia (Gharbi et al., 2013)	Siliana	Sheep	ELISA	3/166 (1.8)	[62]
Tunisia (Khayeche et al., 2013)	Sousse	Sheep	PCR	4/70 (5.7)	[79]
Tunisia (Boughattas et al., 2014)	Tunis	Young sheep	MAT	83/217 (38.2)	[34]
Tunisia (Boughattas et al., 2014)	Tunis	Adult sheep	MAT	92/125 (73.6)	[34]
Tunisia (Lahmar et al., 2015)	Gafsa	Sheep	MAT	82/204 (40.2)	[86]
Tunisia (Lahmar et al., 2015)	Gafsa	Goats	MAT	11/32 (34.4)	[86]
Tunisia (Lahmar et al., 2015)	Gafsa	Cattle	MAT	3/25 (12)	[86]
Tunisia (Amairia et al., 2016)	Tabarka	Goats	ELISA	17/34 (50)	[9]
Tunisia (Amairia et al., 2016)	Hammam Bourghiba	Goats	ELISA	7/43 (16.3)	[9]
Tunisia (Amairia et al., 2016)	Tabarka	Goats’ milk	PCR	0/34	[9]
Tunisia (Amairia et al., 2016)	Hammam Bourghiba	Goats’ milk	PCR	6/43 (13.9)	[9]
Tunisia (Rouatbi et al., 2017)	Beja	Sheep	PCR	48/150 (32)	[107]
Tunisia (Rouatbi et al., 2017)	Sidi Bouzid	Sheep	PCR	54/174 (31)	[107]
Tunisia (Amdouni et al., 2017)	Beja	Sheep	PCR	50/150 (33.3)	[10]
Tunisia (Amdouni et al., 2017)	Beja	Cattle	PCR	29/150 (19.3)	[10]
Tunisia (Amdouni et al., 2017)	Beja	Goats	PCR	39/120 (32.5)	[10]
Libya (Azwai et al., 1993)	–	Goats	IHAT	−(50)	[14]
Libya (Azwai et al., 1993)	–	Sheep	IHAT	−(26.2)	[14]
Libya (Azwai et al., 1993)	–	Horses	IHAT	−(4.8)	[14]
Libya (Azwai et al., 1993)	An-Najila	Cattle	IHAT	−(27.4)	[14]
Libya (Azwai et al., 1993)	Khadra’	Cattle	IHAT	−(14.3)	[14]
Libya (Azwai et al., 1993)	Al Hany	Cattle	IHAT	−(10.6)	[14]
Libya (El-Gomati et al., 2010)	Tripoli	Mice	Toxocell latex test	21/60 (35)	[49]
Libya (Al-mabruk et al., 2013)	Western, Central, Eastern and Southern	Sheep	LAT^a^	4122/5806 (71)	[8]
Egypt (Rifaat et al., 1976)	Cairo	Chickens	DT^b^	17/85 (20)	[105]
Egypt (Rifaat et al., 1976)	Cairo	Rabbits	DT	49/100 (49)	[105]
Egypt (Ibrahim et al., 1997)	Tanta	Sheep	IHAT	114/258 (44.2)	[72]
Egypt (Ibrahim et al., 1997)	Tanta	Sheep	IFAT	126/258 (48.8)	[72]
Egypt (Hilali et al., 1998)	–	Camels	DAT^c^	29/166 (17.5)	[71]
Egypt (El-Ghaysh, 1998)	Monofia	Donkeys	ELISA	79/121 (65.3)	[48]
Egypt (Dubey et al., 2003)	Giza	Free range chickens	MAT	49/121 (40.5)	[42]
Egypt (Ghazy et al., 2007)	–	Horses	ELISA	160/420 (38.1)	[63]
Egypt (Shaapan et al., 2008)	Cairo	Sheep	MAT	131/300 (43.7)	[112]
Egypt (Shaapan et al., 2008)	Cairo	Sheep	ELISA	125/300 (41.7)	[112]
Egypt (Shaapan et al., 2008)	Cairo	Sheep	IFAT	111/300 (37)	[112]
Egypt (Shaapan et al., 2008)	Cairo	Sheep	DT	102/300 (34)	[112]
Egypt (Ibrahim et al., 2009)	Sharkia	Cattle	ELISA	10/93 (10.7)	[73]
Egypt (Haridy et al., 2010)	Cairo	Working donkeys	ELISA	45/100 (45)	[69]
Egypt (Haridy et al., 2010)	Cairo	Donkeys’ milk	ELISA	7/15 (46.7)	[69]
Egypt (Ghoneim et al., 2010)	El Fayoum	Sheep	ELISA	61/62 (98.4)	[64]
Egypt (Ghoneim et al., 2010)	El Fayoum	Sheep	PCR	42/62 (67.7)	[64]
Egypt (Ghoneim et al., 2010)	El Fayoum	Goats	ELISA	10/24 (41.7)	[64]
Egypt (Ghoneim et al., 2010)	El Fayoum	Goats	PCR	6/24 (25)	[64]
Egypt (Harfoush and Tahoon, 2010)	Kafr El-Sheikh	Domestic ducks	IHAT	−(55)	[68]
Egypt (Harfoush and Tahoon, 2010)	Kafr El-Sheikh	Free-range chickens	IHAT	−(38.1)	[68]
Egypt (Harfoush and Tahoon, 2010)	Kafr El-Sheikh	Turkeys	IHAT	−(29.4)	[68]
Egypt (Ashmawy et al., 2011; Harfoush and Tahoon, 2010)	Kafr El-Sheikh	Domestic rabbits	IHAT	−(17.5 to 37.5)	[12, 68]
Egypt (Al-Kappany et al., 2011)	Cairo	Feral cats	MAT	172/180 (95.5)	[7]
Egypt (Ashmawy et al., 2011)	Alexandria	Domestic rabbits	IHAT	9/85 (10.6)	[12]
Egypt (Ashmawy et al., 2011)	Behera	Domestic rabbits	IHAT	6/69 (8.7)	[12]
Egypt (Barakat et al., 2012)	Cairo	Chickens	ELISA	−(62.2)	[18]
Egypt (Barakat et al., 2012)	Gharbia	Chickens	ELISA	−(82.3)	[18]
Egypt (Barakat et al., 2012)	Kafr El sheikh	Chickens	ELISA	−(67.1)	[18]
Egypt (Barakat et al., 2012)	Quena	Chickens	ELISA	−(75)	[18]
Egypt (Barakat et al., 2012)	Sharkia	Chickens	ELISA	−(59.5)	[18]
Egypt (Barakat et al., 2012)	Sohag	Chickens	ELISA	−(50)	[18]
Egypt (Behairy et al., 2013)	Giza	Turkeys	MAT	103/173 (59.5)	[45]
Egypt (Behairy et al., 2013)	Giza	Chickens	MAT	51/108 (47.2)	[45]
Egypt (Behairy et al., 2013)	Giza	Ducks	MAT	24/48 (50)	[45]
Egypt (El-Madawy and Metawea, 2013)	Ismailia	Ostriches	ELISA	15/120 (12.5)	[51]
Egypt (El-Madawy and Metawea, 2013)	Ismailia	Ostriches	PCR	9/120 (7.5)	[51]
Egypt (Behairy et al., 2013)	Giza	Dogs	MAT	50/51 (98)	[45]
Egypt (Behairy et al., 2013)	Giza	Hearts of dogs	Bioassay	22/43 (51.2)	[45]
Egypt (Ahmed et al., 2014)	Sharkia	Sheeps’ milk	PCR	1/50 (2)	[4]
Egypt (Ahmed et al., 2014)	Sharkia	Goats’ milk	PCR	4/50 (8)	[4]
Egypt (Ahmed et al., 2014)	Sharkia	Cows’ milk	PCR	0/50	[4]
Egypt (Mahmoud et al., 2015)	Gharbia	Stray cats	IHAT	17/92 (18.5)	[90]
Egypt (Mahmoud et al., 2015)	Gharbia	Stray cats	IFAT	19/92 (20.7)	[90]
Egypt (Mahmoud et al., 2015)	Gharbia	Owned cats	IHAT	4/32 (12.5)	[90]
Egypt (Mahmoud et al., 2015)	Gharbia	Owned cats	IFAT	5/32 (15.6)	[90]
Egypt (Ibrahim et al., 2017)	Menoufia	Sheep	ELISA	−(51.04)	[74]
Egypt (Ibrahim et al., 2017)	Menoufia	Sheep	RT-PCR	−(17.71)	[74]
Egypt (Ibrahim et al., 2017)	Gharbia	Sheep	ELISA	−(52.70)	[74]
Egypt (Ibrahim et al., 2017)	Gharbia	Sheep	RT-PCR	−(17.57)	[74]

a **LAT:** Latex Agglutination Test.

b **DT:** Dye Test.

c **DAT:** Direct Agglutination Test.

### *Toxoplasma gondii* infection in Algeria

The first paper studying *T. gondii* in Algeria was published in 1955 by Balozet [16]. It was a serological investigation (complement-fixation test) confirming the presence of *T. gondii* antibodies in 10% and more than 30% in humans and dogs, respectively. Schneider et al. [110] found higher infection rates in patients (53.2%) by the indirect immunofluorescence test. All other published papers concerned seroprevalence in pregnant women. Serological surveys revealed small variations in infection rates: Messerer et al. [93] found the same results in Annaba (47.8%), whereas Berredjem et al. [31] found lower seroprevalence (39.8%). The combination between serological and molecular tools for congenital toxoplasmosis diagnosis was only studied by Berredjem et al. [31]. The results of this study should be interpreted with caution since using PCR for the detection of the parasite’s DNA in peripheral blood samples has low sensitivity and is indicated only in certain cases of immunocompromized patients [5]. Consumption of undercooked meat and the presence of cats in the household were the major risk factors associated with *T. gondii* infection [31]. Genotype II was isolated from a congenital toxoplasmosis case and human strains [15].

**Figure 2:**
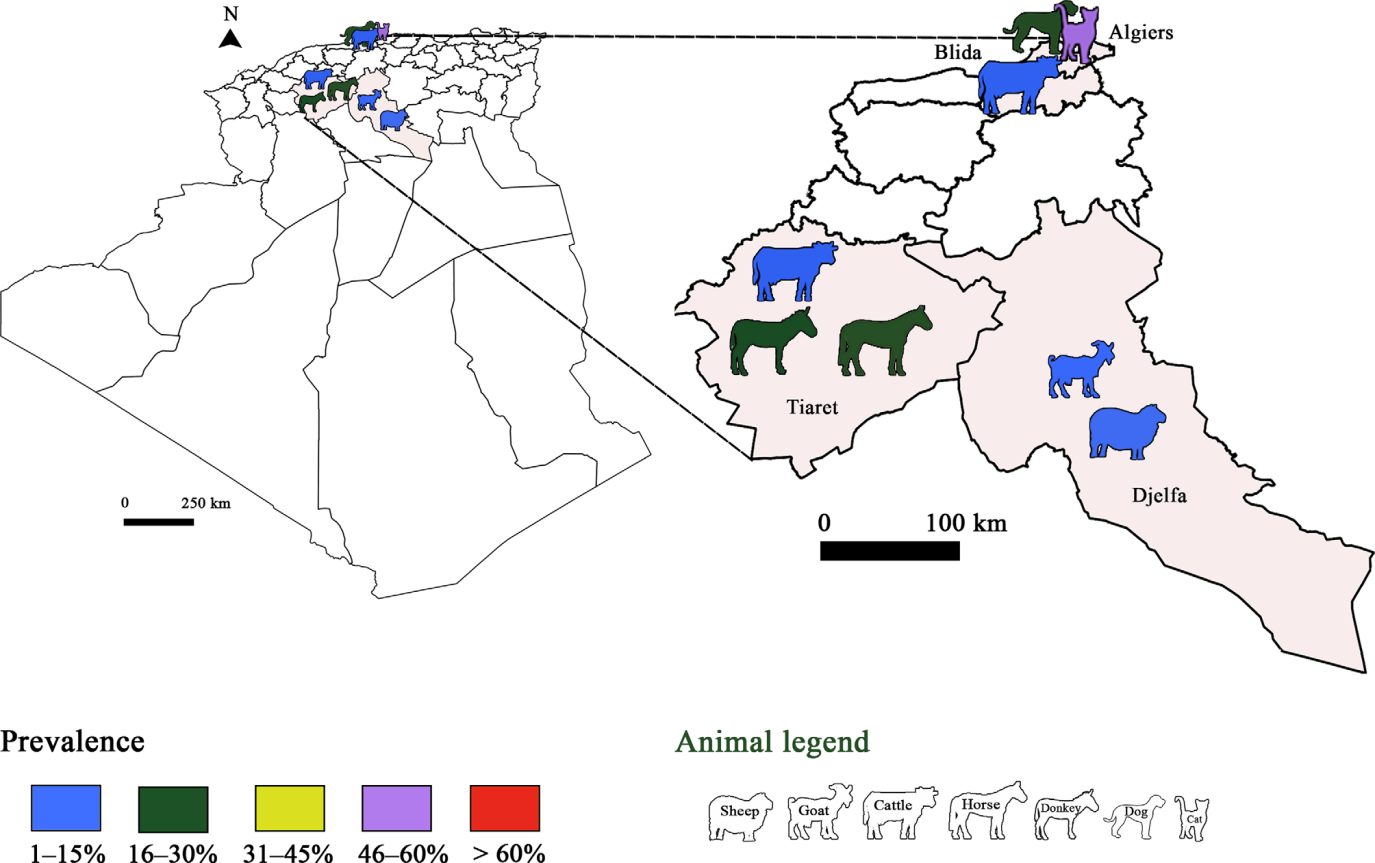
Prevalence of *Toxoplasma gondii* infection in Algeria: Animal toxoplasmosis, seroprevalence.

Only serological studies have been performed to detect *T. gondii* in animals in Algeria (Fig. 2). Seroprevalence rates of *T. gondii* in sheep were low (8.28%) in Eastern and South Algeria, and in Djelfa locality (11.6%) using ELISA and immunofluorescent antibody tests (IFAT), respectively [36, 37]. Through these studies, three risk factors were associated with *T. gondii* infection, namely season, origin of animals, and absence of abortion history. Concerning the season, summer, spring and autumn were characterized as more suitable periods for oocyst survival. The authors also suggested that the high relative humidity that typifies Northern Algeria (Center, Eastern and Western) enhances oocyst viability since infection rates were higher than in the south. The presence of *T. gondii* antibodies in goats has only been investigated using IFA testing in a study carried out by Dechicha et al. [37]. In the same study, the seroprevalence of *T. gondii* in cattle was lower than that reported for sheep and goats. This emphasises the lower susceptibility of cattle to *T. gondii* compared to small ruminants. Other studies were conducted in cats, horses and donkeys. The seroprevalence in cats was comparable to that in humans, and the two species shared the same genotype, i.e. genotype II [93, 129].

### *Toxoplasma gondii* infection in Tunisia

The first study concerning human infection was published in 1970 followed by many other studies in humans [80, 81], or studies of *T. gondii* as a parasite of food origin [82]. In the first survey, conducted by Ben Rachid and Blaha [30] using the Sabin-Feldman Dye Test (SFDT), results showed that the overall seroprevalence of *T. gondii* infection increased with age. This pattern was later confirmed in a larger survey including 142 individuals from the northern parts of the country using ELISA and IFAT [35]. This epidemiological profile suggests that even though infection is a frequent event in early childhood, women of childbearing age remain susceptible to toxoplasmosis. Other surveys focused on pregnant women and congenital toxoplasmosis [19, 25, 114]. The seroprevalence rates range from 39.3% in the southern regions to 47.7% in the northern regions using ELISA [26, 56, 111]. Most of the Tunisian authors suspected consumption of undercooked meat and unwashed vegetables as the two main contamination routes [56]. Infection was present in regions where agriculture was the predominant activity and sheep meat was the most consumed meat [30]. Toxoplasmosis is regularly diagnosed in immunocompromized patients in Tunisia. Before highly active antiretroviral therapy (HAART), cerebral toxoplasmosis was reported as one of the most prevalent infections in patients with HIV [111].

**Figure 3:**
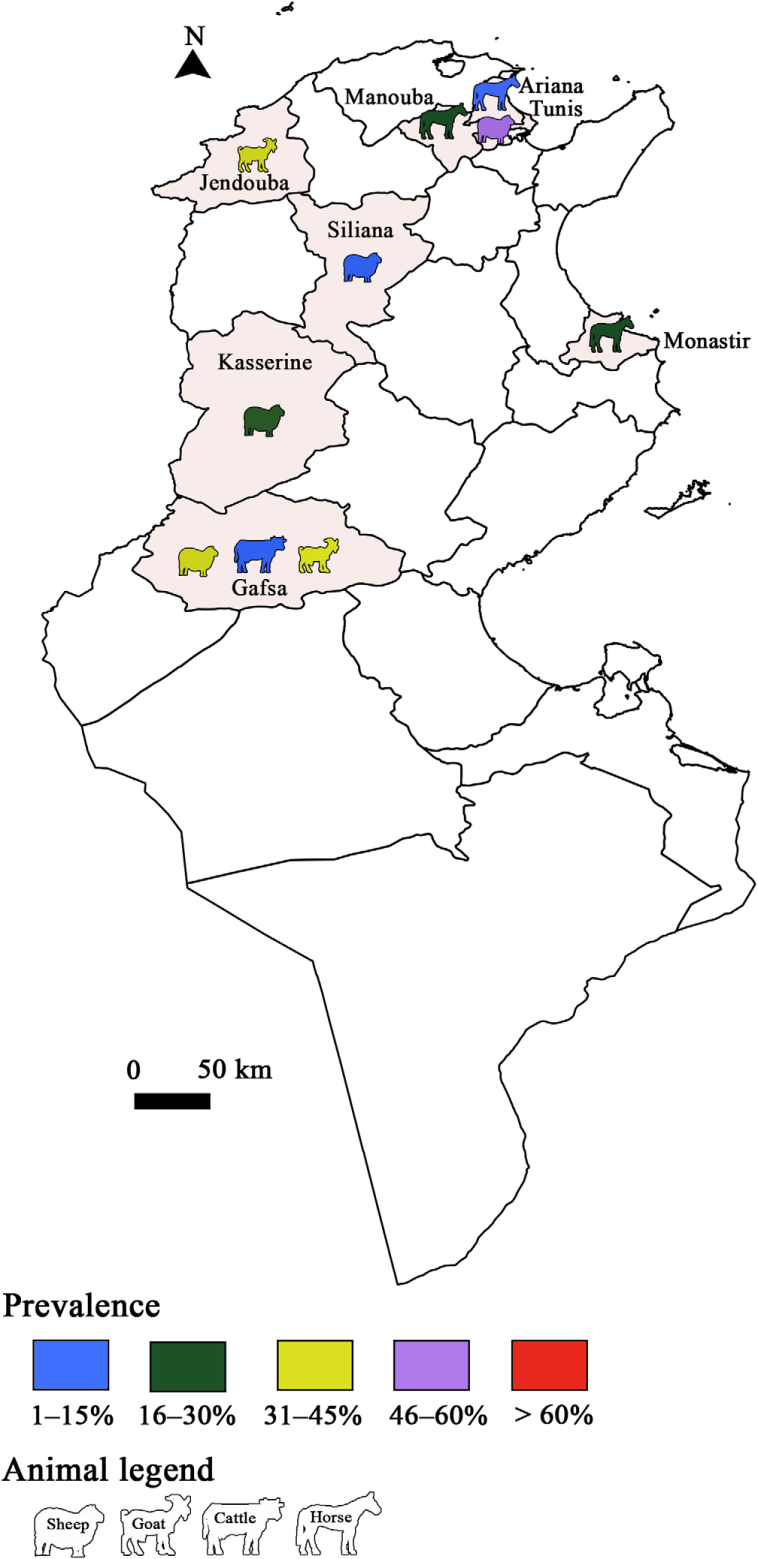
Prevalence of *Toxoplasma gondii* infection in Tunisia: Animal toxoplasmosis, seroprevalence.

**Figure 4:**
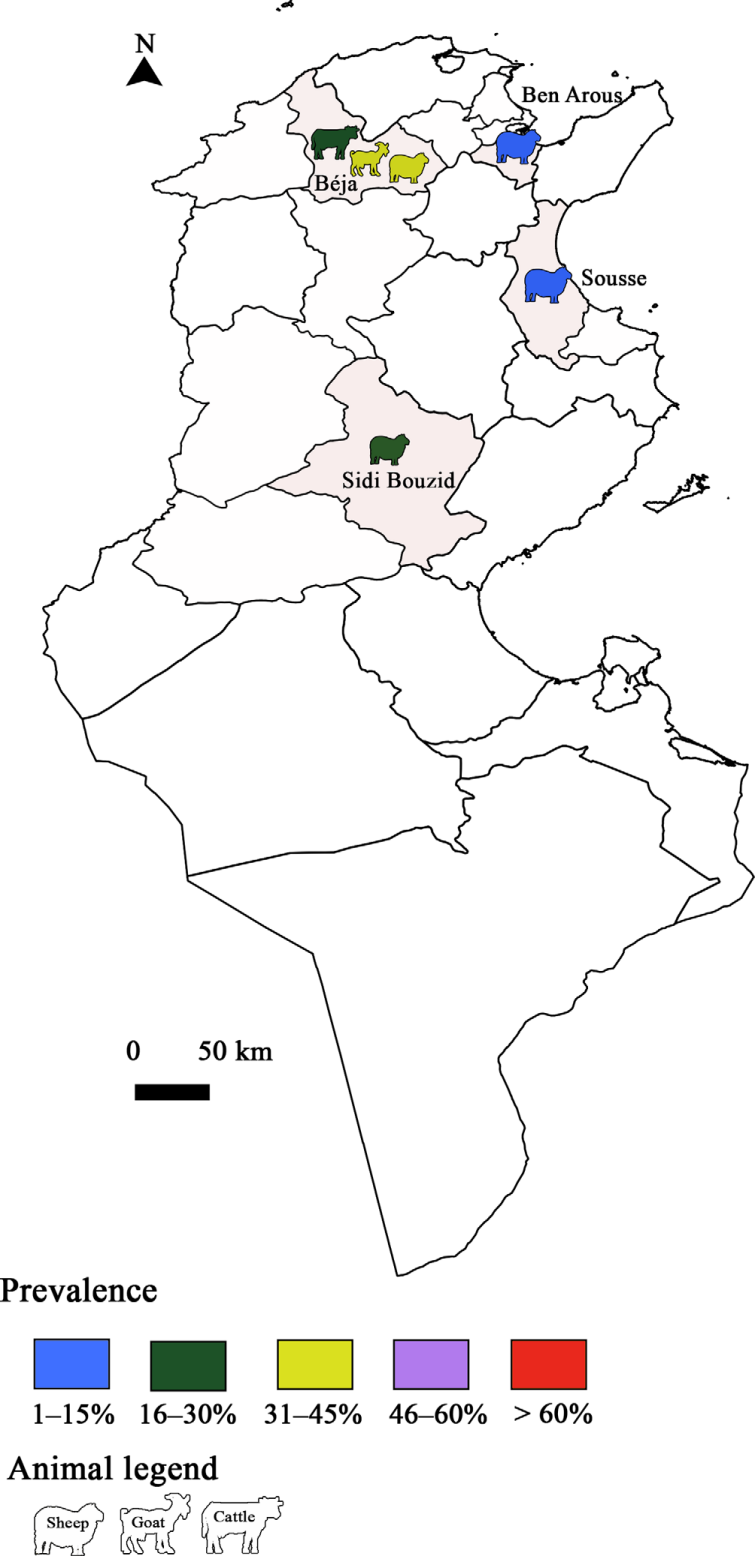
Prevalence of *Toxoplasma gondii* infection in Tunisia: Animal toxoplasmosis, molecular prevalence.

*Toxoplasma gondii* infection has also been studied in animals and several studies were conducted in sheep (Figs. 3 and 4). Sero-surveys found a maximum infection rate of 73.6% using a modified agglutination test (MAT) [34, 62, 86]. Using molecular tools, Boughattas et al. [34] found the highest infection rate in ewe tissues (50%) in Tunis city. Consequently, the authors considered sheep meat as a major risk factor of *T. gondii* transmission through meat consumption. Three studies determined the molecular prevalence of *T. gondii* in apex heart samples from sheep, and the infection rates ranged between 5.7% and 25.5% [62, 79] in the first two studies. The lowest rate was reported by Khayeche et al. [79] in the third study, where apex hearts samples were collected from slaughtered sheep during the Muslim feast of Eid Al-Adha. This could be explained by the very low age of these animals. In fact, the majority of households (92.9%) slaughtered a sheep aged less than 1 year. This study highlighted that the majority of meat handlers did not respect hygiene rules, since 91% of them did not wash their hands after handling and before preparing or consuming food. In addition, the presence of factors that increase the risk of toxoplasmosis such as cat feces or eating raw meat during Eid Al-Adha was detected in 14% of the households.

Along with sheep, *T. gondii* infection was studied in other species such as goats and horses [10, 33]. To assess the risk of toxoplasmosis transmission through contaminated food, one study was conducted in goat milk samples, reporting a molecular infection rate of 7.8% [9]. Detecting parasite DNA in milk does not mean that the parasite is alive; further studies are needed to determine parasite viability. Additionally, it has been confirmed experimentally that tachyzoites survive in goat’s milk for three to seven days at +4 °C [116]. Moreover, Tenter et al. [120] confirmed that unpasteurized goat’s milk is an important source of human toxoplasma infection.

### *Toxoplasma gondii* infection in Libya

The first serological survey on human infection was carried out by Khadre and El Nageh [78] in Tripoli. In the same locality (Tripoli), Gashout et al. [61] reported an infection rate of 38.5% in women with spontaneous abortions. Positive results were also detected in 47.7% of pregnant women in Benghazi (by indirect hemagglutination antibody testing (IHAT)), with the highest rate observed among the older age group (63.3%) [77]. Lower seroprevalence in pregnant women was found in the Alkhoms district, using ELISA (39.3%) [60]. The authors of this study emphasized an association between *T. gondii* prevalence and several risk factors, including age (age group), living area (rural and urban), diet (consumption of lamb meat), drinking water source, and contact with cats. Recently, *T. gondii* DNA was detected in 9.9% of the umbilical cord of neonates, indicating a high level of congenital toxoplasmosis [67]. In 2014, the prevalence of *T. gondii* infection in psychiatric patients in Tripoli was estimated and this prevalence was significantly higher than in the control group. The authors explain this as a causal relationship between toxoplasmosis infection and psychiatric diseases [53]. Nevertheless, this result needs to be investigated further to explain the relationship between toxoplasmosis and psychiatric diseases.

**Figure 5:**
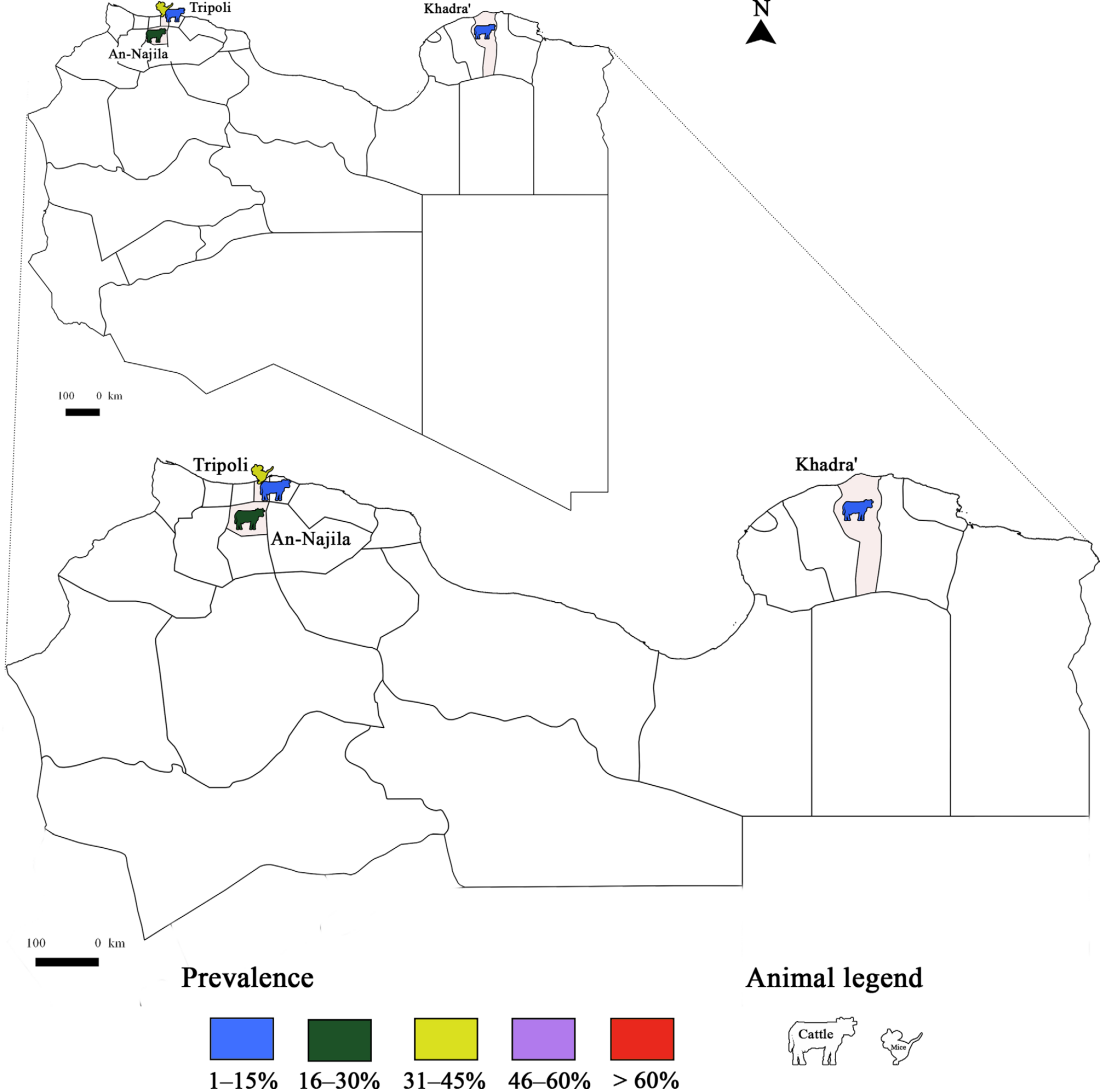
Prevalence of *Toxoplasma gondii* infection in Libya: Animal toxoplasmosis seroprevalence.

To the best of our knowledge, there are few studies in Libya related to *T. gondii* infection in animal species (Fig. 5). Indirect hemagglutination testing (IHAT) was used to serologically determine the prevalence of the infection in cattle, sheep, goats and horses sampled from different parts of Libya by Azawi et al. [14]. A higher seroprevalence (76.6%) was reported in sheep from the western region compared to the central region using latex agglutination testing (LAT). The presence of cats and the specific climate in each region were the two determinants of *T. gondii* infection distribution. Associations with different risk factors were studied, showing a significant correlation with the age group, abortion in sheep, and the management system (extensive and intensive) [8].

### *Toxoplasma gondii* infection in Egypt

Toxoplasmosis was declared for the first time as a “new disease” in Egypt in 1952 [108]. Egyptian studies on *T. gondii* infection exceed 200 articles and clearly show it is widespread in the country. Nevertheless, many studies were not taken into consideration to respect the imposed criteria in this review. The seroprevalence of *T. gondii* in humans is high, reaching 59.6% [54, 72, 130], and both *T. gondii* genotypes I and II were reported in Egyptian patients [3]. *T. gondii* DNA was detected in several population groups: in children and their mothers (43.7% and 37.5%, respectively), and in inhabitants and workers [13, 72, 130]. Pregnant women, being the main category at risk, had high *T. gondii* infection rates [47, 50, 73, 76]. The seroprevalence of *T. gondii* in Egyptian cats reached 97% [6, 7]. Since the majority of cats were seropositive, the soil is suspected to be heavily contaminated with oocytes. This supports the idea that contact with cats might be the main risk factor for toxoplasmosis transmission in Egypt.

**Figure 6:**
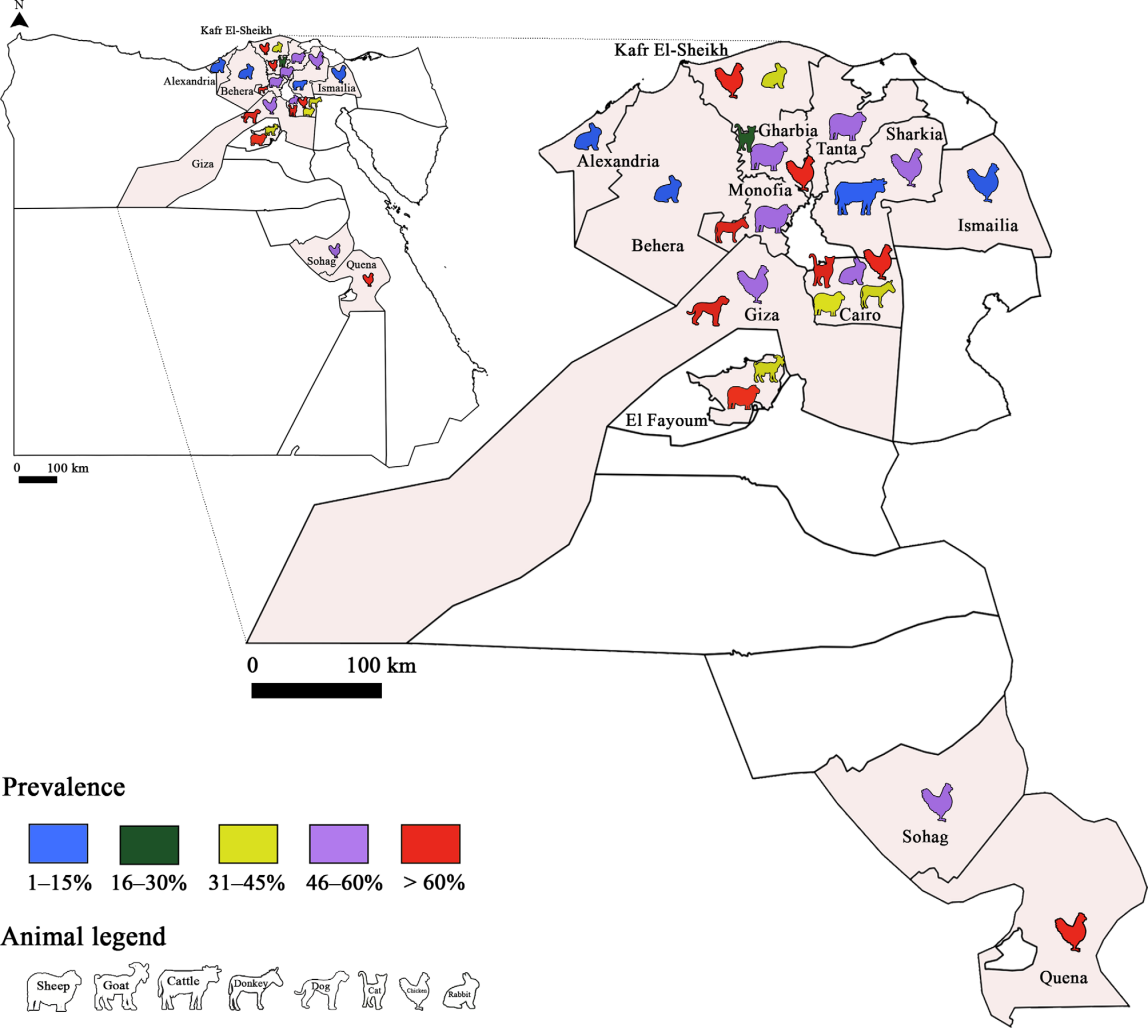
Prevalence of *Toxoplasma gondii* infection in Egypt: Animal toxoplasmosis, seroprevalence.

**Figure 7:**
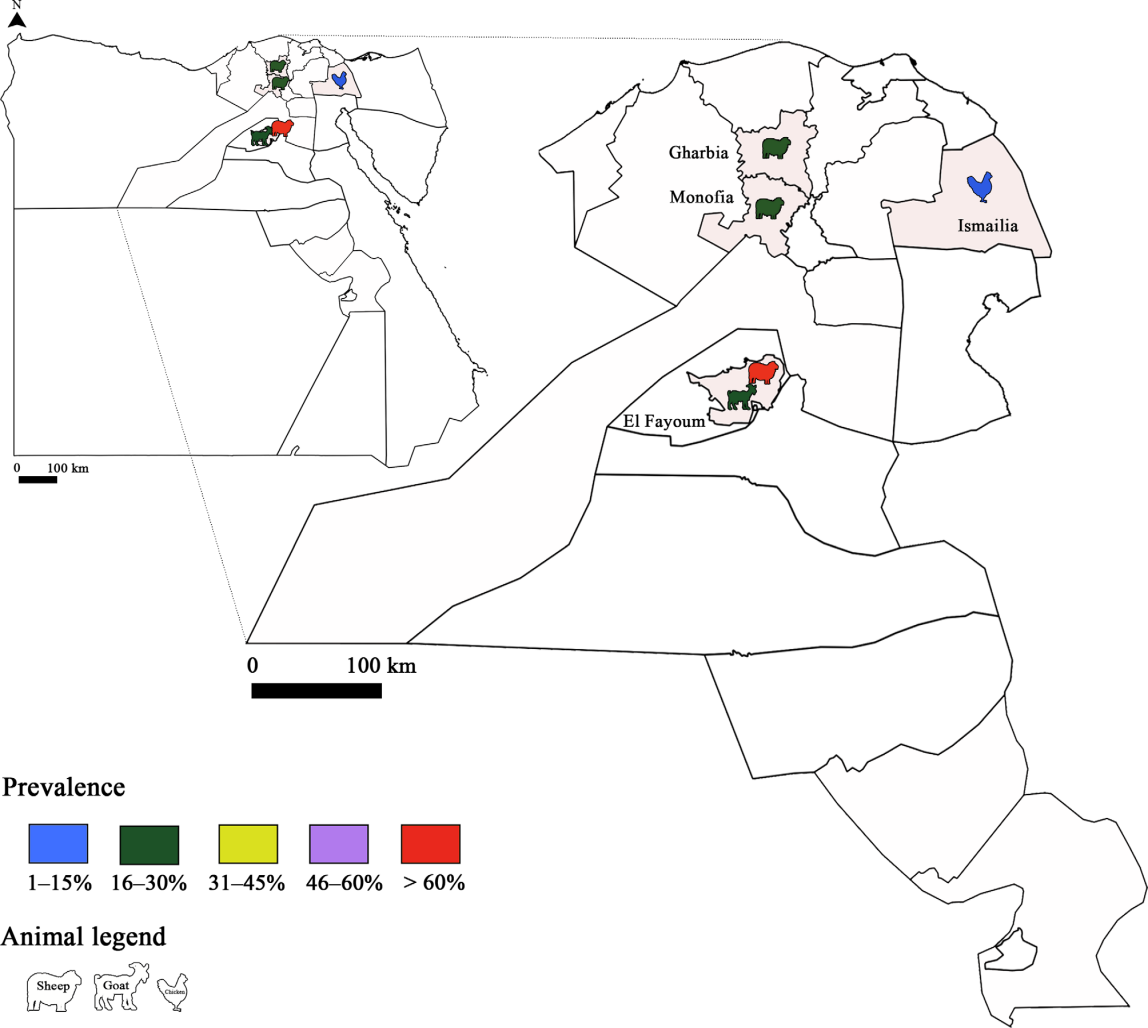
Prevalence of *Toxoplasma gondii* infection in Egypt: Animal toxoplasmosis, molecular prevalence.

In small ruminants, high prevalence of infection was reported, reaching 98.4% and 41.7% by serological tools, and 67.6% and 25% by molecular methods in sheep and goat samples, respectively [64] (Figs. 6 and 7). The infection rate with *T. gondii* in sheep has shown a downward trend in recent studies, at 17.65% [74]. This could be explained by the sensitivity of the tests used, such as real-time PCR. Also, a combination of techniques (serological and molecular methods) increases sensitivity and specificity. In Egyptian cattle, *T. gondii* prevalence was comparably low. *Toxoplasma gondii* in chickens, ostriches and ducks was also reported [18, 42, 51]. Isolates of *T. gondii* from chicken samples belonged to genotypes II and III, while in ducks, the genotype was type III. Several studies were performed in camels, horses, donkeys and stray dogs [45, 63, 69, 71]. Recently, a prevalence study in milk samples from cows, sheep and goats showed rates of 0%, 2% and 8%, respectively [4].

## Discussion: overall analyses of the surveys carried out in North Africa

Toxoplasmosis is a widespread zoonosis in North African countries in both humans and animals. This zoonosis was studied in five North African countries (Morocco, Algeria, Tunisia, Libya and Egypt), demonstrating that the epidemiological cycle of this protozoan is very well maintained in this region.

**Figure 8:**
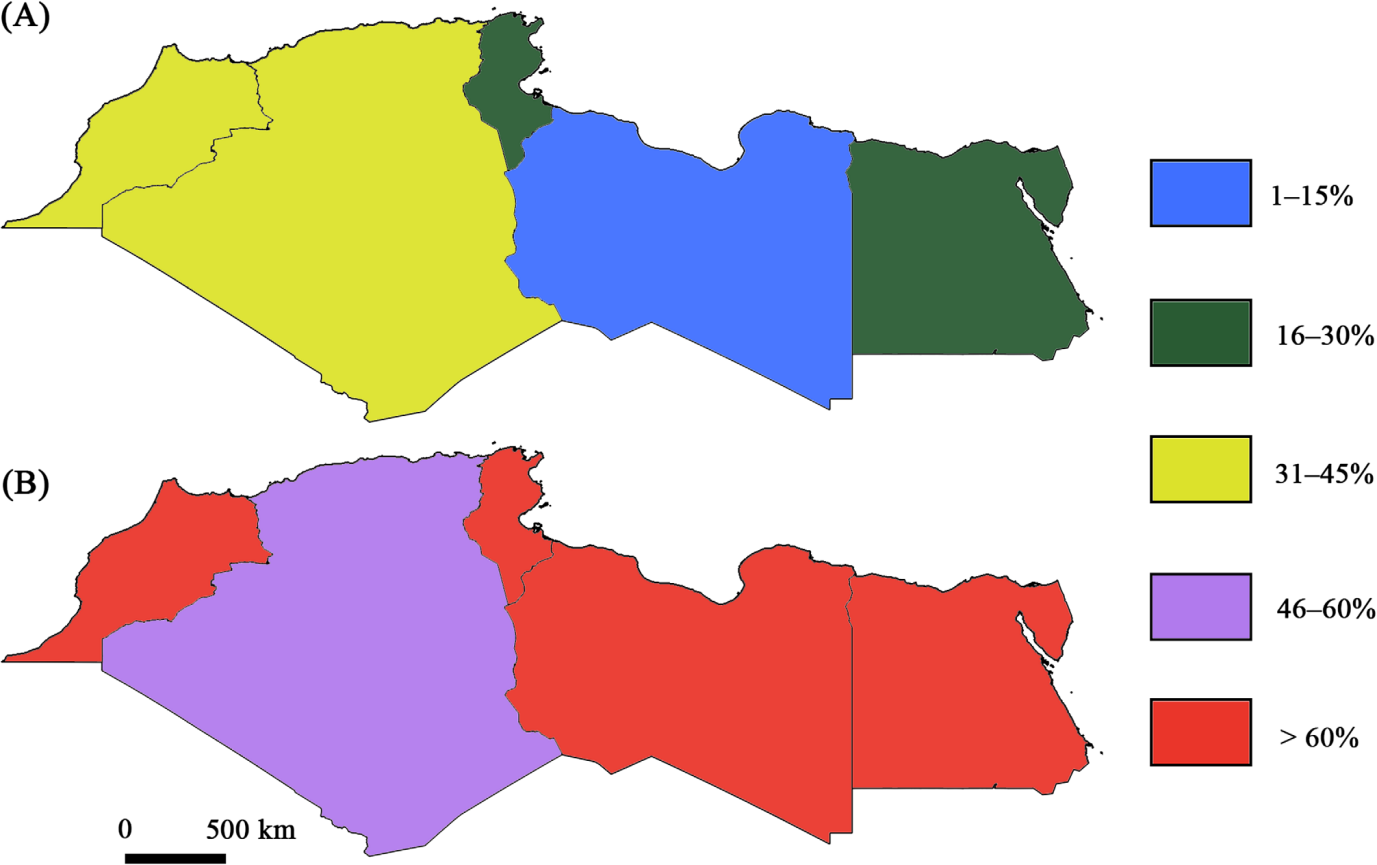
Overall status of *Toxoplasma gondii* seroprevalence in humans in North African countries: (A) Minimum infection rates; (B) Maximum infection rates.

**Figure 9:**
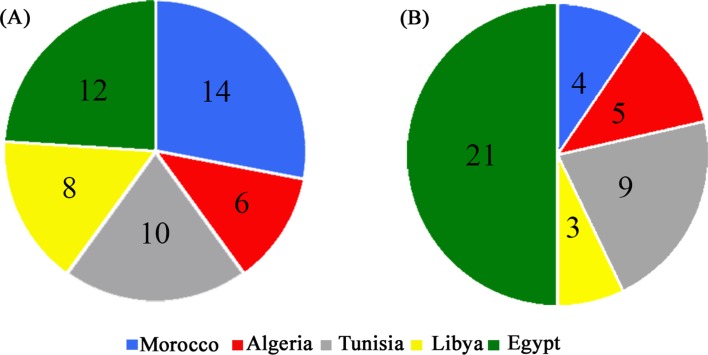
Overall number of studies dealing with human and animal toxoplasmosis in five North African countries (Morocco, Algeria, Tunisia, Libya and Egypt).

**Figure 10:**
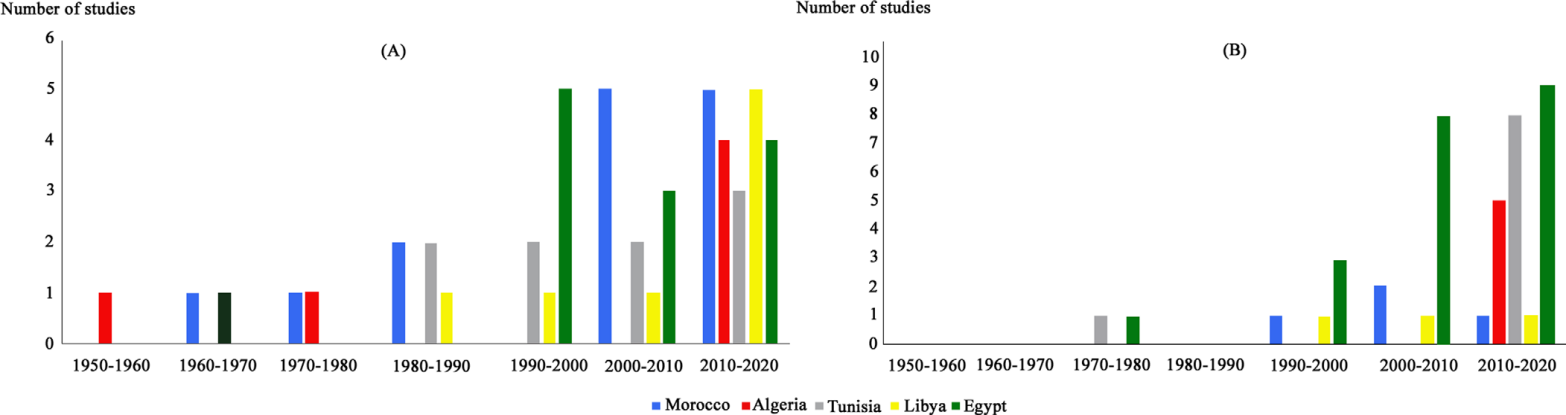
Distribution of studies dealing with human and animal toxoplasmosis in five North African countries over time (Morocco, Algeria, Tunisia, Libya and Egypt).

The number of studies dealing with *T. gondii* infection in humans was almost equal in the five countries (Fig. 8). For animal infections, more studies were performed in Egypt (Fig. 9). For human and animal infections and at the region level, a gap of knowledge was recorded during the period between 1960 and 1970. An increase in the number of studies was recorded in the period between 2000 and 2018, with a marked predominance of studies conducted in Egypt (Fig. 10). This is may be due to the recognition that toxoplasmosis is a significant public health challenge and to public awareness. In Morocco for example, awareness about the risk of this zoonosis is increasing thanks to new initiatives and research established by the Ministry of Health [46].

Morocco’s health policy, like in Tunisia and Algeria, is influenced by French approaches that represent a strong benchmark for countries planning to set up a national program against toxoplasmosis [46]. In fact, France reported a decrease in the prevalence and severity of toxoplasmosis after the implementation of mandatory gestational screening, with standardized screening and treatment protocols in addition to public awareness.

To detect *T. gondii* infection, many techniques were used and varied widely between the five countries. In general, there was a lack of diversity in techniques and only serological methods were used to detect human and animal infection. This could be explained by the lack of molecular diagnostic tools especially in state laboratories, and the limited information regarding contributing risk factors [46].

For human infection, there was broad diversity in sampling in the five countries. Only in Egypt, a variety of animal species was screened for the presence of *T. gondii* (turkeys, ducks, chicken, ostriches, sheep, goats, etc.). This is a concern since *T. gondii* infection is widespread in food animals consumed in North Africa, especially chicken, camel, sheep, and goat meat [123]. In both human and animal *T. gondii* infection, there was heterogeneity in the molecular and serological prevalences estimated in the five North African countries, which is correlated with the techniques used. In fact, serological methods appear to lack sensitivity and specificity, even though the qualitative detection of antibodies remains a standard tool. At the same time, there are differences within the serological techniques. Moreover, Dubey et al. [39] found that the diagnostic performance of a MAT was higher than that of ELISA.

In many studies conducted in the five countries of North Africa, age was a major risk factor. Higher levels of positive results were found in older animals. This is consistent with many studies conducted in France and Iran for example [44, 66]. The higher prevalence in adult compared to younger animals may be explained by the longer period of exposure [123].

Farm management is also a risk factor. For example, in Algeria, sheep are reared in extensive systems and fed on fresh bulk feed or pasture, which are a greater risk as sources of contamination [36].

Generally, in North Africa, trends indicate that production systems have become more intensive: agricultural by-products, non-conventional sources of feed, and commercial concentrates are increasingly used. The use of concentrates represents a risk factor since contaminated grain could be responsible for the rapid spread of infection in a flock [101]. Widespread oocyst contamination in the environment is also due to fecal contamination of soil and groundwater by either domestic or feral cats [123].

Since animals play an important role in the transmission of *T. gondii* to humans via meat or milk consumption, or by the prominent role of cats in the contamination of the environment by oocysts, studying prevalence rates of animal toxoplasmosis will be helpful to estimate the rate of human toxoplasmosis [66].

In the five North African countries studied, little is known about the epidemiology of toxoplasmosis in wild animals. In fact, there is no information about *T. gondii* prevalence in wild felids.

Concerning the evolutionary history of *Toxoplasma*, the presence of *T. gondii* in Africa could be due to the spread of this parasite from the Americas to Asia via the Bering Strait. It is believed that this parasite entered Africa around 1.5 million years ago [20]. Only genotypes II and III have been identified in North Africa among the three archetypal lineage types I, II, and III. Apart from their presence in North Africa, types II and III are the main lineages in the Middle East, Europe, and North America [59].

The similarities in *T. gondii* infection patterns between the populations of the Mediterranean basin could be explained by human travel and trade within these regions [59, 91]. Moreover, the emergence of clonal lineages II and III coincided with the advent of agriculture 10,000 years ago and cat domestication in the eastern Mediterranean basin [21, 118, 124].

In North African countries, no specific national programs against toxoplasmosis are currently in place. The major tool for avoiding congenital *T. gondii* infections and their complications is prevention. The preventive measures depend strongly on the knowledge of women about toxoplasmosis. However, this remains a major problem in the North African context since, within the same country, there are considerable differences in the socioeconomic status of women.

Even though serological screening for the infection is highly recommended during the first antenatal care visit, an analysis of the current situation indicates that a control program for human toxoplasmosis is lacking and pregnant women are not sufficiently aware of all the infection routes.

## Conclusion

Toxoplasmosis represents a significant health threat to both humans and livestock, inducing high morbidity and economic losses. While the occurrence of *T. gondii* is fairly well documented in most countries, little information is available to quantify the resulting impact for the livestock sector and for public health. Having better impact data would make it easier to convince decision makers to invest in toxoplasmosis control and prevention. In addition, more in-depth epidemiological studies are needed to inform the design of regional strategies and to guide implementation of control programs involving both the medical and veterinary sectors.

Given the involvement of the environment in the transmission cycle, attention should also be given to environmental sampling in order to develop adequate transmission models between animals, the environment and people, providing the basis for a real One Health approach in the control of toxoplasmosis.
